# Silk physico-chemical variability and mechanical robustness facilitates intercontinental invasibility of a spider

**DOI:** 10.1038/s41598-019-49463-9

**Published:** 2019-09-13

**Authors:** Carmen Viera, Luis F. Garcia, Mariángeles Lacava, Jian Fang, Xungai Wang, Michael M. Kasumovic, Sean J. Blamires

**Affiliations:** 1 Entomología, Universidad de la República de Uruguay, Montevideo, Uruguay; 2 Laboratorio Ecología del Comportamiento (IIBCE), Montevideo, Uruguay; 3 Centro Universitario Regional del Este, Sede Treinta y Tres, Universidad de la República, Treinta y Tres, Uruguay; 4 Centro Universitario de Rivera, Universidad de la República, Rivera, Uruguay; 5 Deakin University, Institute for Frontier Materials (IFM), Waurn Ponds Campus, Geelong, 3220 Australia; 6 Evolution & Ecology Research Centre, School of Biological, Earth & Environmental Sciences, The University of New South Wales, Sydney, NSW 2052 Australia

**Keywords:** Ecology, Invasive species

## Abstract

There are substantive problems associated with invasive species, including threats to endemic organisms and biodiversity. Understanding the mechanisms driving invasions is thus critical. Variable extended phenotypes may enable animals to invade into novel environments. We explored here the proposition that silk variability is a facilitator of invasive success for the highly invasive Australian house spider, *Badumna longinqua*. We compared the physico-chemical and mechanical properties and underlying gene expressions of its major ampullate (MA) silk between a native Sydney population and an invasive counterpart from Montevideo, Uruguay. We found that while differential gene expressions might explain the differences in silk amino acid compositions and protein nanostructures, we did not find any significant differences in silk mechanical properties across the populations. Our results accordingly suggest that *B*. *longinqua*’s silk remains functionally robust despite underlying physico-chemical and genetic variability as the spider expands its range across continents. They also imply that a combination of silk physico-chemical plasticity combined with mechanical robustness might contribute more broadly to spider invasibilities.

## Introduction

Invasive species successfully establish themselves into new environments upon arrival and impact local ecosystems^1,2^, with many having negative effects on native organisms^3–5^. They are most commonly introduced to these new environments by human movements and trade^2^.

Phenotypic variations, which may be a consequence of a variable genetic expression or by environmental factors directly affecting certain phenotypic features, can facilitate organismal adaptations to novel environments^6,7^. For instance, trees with woody structures that can adjust biomechanically to the physical demands of different environmental stressors are more successful invaders than plants with structures that cannot readily adjust^8^. Phenotypic variability in itself, nonetheless, is an evolutionary character that varies among species^9^. Consequently, the invasive success of an animal is a complex interplay between trait properties, the underlying genetics, and a multitude of environmental factors that interact with genotypic and phenotypic expressions^6,8–10^. Accordingly, the capacity of an organism for invasion, i.e. its “invasibility”^8^, may vary over space and time, making predicting invasions and the likely invaders exceptionally difficult for any given place or moment.

**Figure 1:**
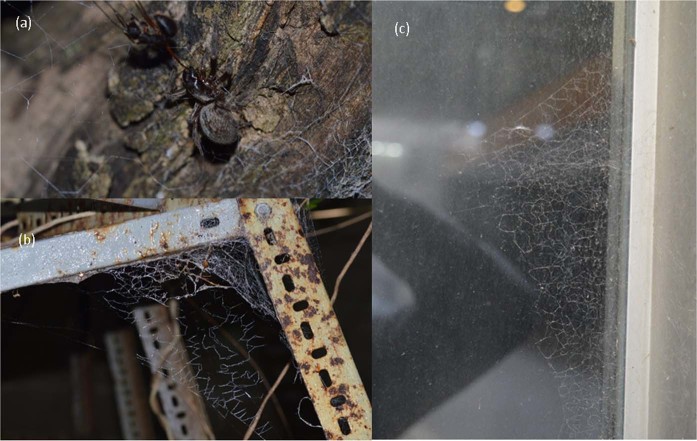
Photographs of (**a**) *Badumna longinqua* consuming an ant in Montevideo, and *B*. *longinqua* webs in (**b**) Montevideo and (**c**) Sydney (all photographs by S. Blamires). Shows the typical microhabitats at the urban locations where they spiders were collected.

The brown house spider, *Badumna longinqua*, (family Desidae) inhabits urban and rural habitats in eastern and southwestern Australia^11,12^. They are distinguishable by their flat, relatively large sheet webs containing dense, irregular lines of cribellate (i.e. lacking glue) capture silk^13^, which they often build on vegetation and urban structures (see photographs in Framenau *et al*.^12^; Pompozzi *et al*.^14^; Simó *et al*.^15^, and Fig. 1). This species is an exceptionally good invader of new environments, having successfully invaded New Zealand, much of North and South America, and parts of Europe^14–16^. They nonetheless appear to not have experienced any significant external morphological changes as they move across different environments^14–18^ (SJB personal observation). Whether or not their silk properties and the underlying genetic expressions vary as they invade new environments, nevertheless, remains unknown.

*B*. *longinqua* was introduced into Uruguay in the 1850s with the importation of *Eucalyptus* trees from Australia. It remained relatively obscure for some time, but in the early 1900s became increasingly visible in rural and urban areas throughout the country^18^. The effects of *B*. *longinqua* invasion on native Uruguayan spiders and insects have not been documented, but we have observed them invading and destroying colonies of the subsocial spider *Anelosimus vierae* within trees in urban Montevideo, with coincidental declines in the size and density of these colonies (see Supplementary Fig. S1).

Montevideo, Uruguay, and Sydney, Australia, are at a similar latitude (around 34–35° South), have similar maximum and minimum summer and winter temperatures (Supplementary Fig. S2a), but differ in relative humidity (with Montevideo exceeding Sydney all year round, see Supplementary Fig. S2b). While a full dietary survey is yet to be undertaken, we have passively seen *B*. *longinqua* consume ants, small flies, and small beetles at both locations. However, only in Montevideo have we seen them attack and consume other spiders, primarily *A*. *vierae* colonies within trees. The attacking of *A*. *vierae* colonies by *B*. *longinqua* thus seems to represent a behavioural and/or dietary shift in the Montevideo population compared to the Sydney population.

Spiders are particularly interesting to study with regard to their invasibilities because they secrete a material that may be considered an extended phenotype, silk^19^, which is constrained by similar selective pressure as other phenotypic features^19–21^. Silk is predominantly used by spiders and insects as components of eggsac cocoons, retreats, and prey catching webs^22^. It is also plays important roles in many behavioural contexts, such as a life line when falling, for interspecific signalling, sperm transfer, sexual communication, nuptial gifts, dispersal, and as a sensory structure^22–25^. An explicit feature of spider silk is that its physico-chemical properties vary across environmental conditions, for instance as humidity, wind, and the spider’s diet changes^22,26–28^, with potential knock-on effects on user fitness^21^. Like somatic features, shifts in spider silk properties can be a consequence of either or both a change in expression of specific genes and the environment directly affecting the structural integrity of silk proteins^29^. Since many studies^22,29–31^ have shown the structural integrity of silk proteins to directly or indirectly influence silk mechanics, it is highly probable that shifts in silk protein structure has consequences for silk mechanical properties.

We consider it appropriate to presume here that since environmental factors affect dragline silk properties across hierarchical levels, encountering new environments will adversely affect silk functionality, which could be potentially problematic for spiders. Nonetheless, many spiders around the world are excellent invaders of novel environments^17,20,32,33^. Perhaps this is because spiders that are adept at invading new environments have less variable silks (i.e. silk with properties that remain stable across environments) than those that do not readily invade new environments. Alternatively, silk variability might act as a means for highly invasive spiders to rapidly adapt to the physical demands of moving into new environments so long as the silks retain their functional integrity. This study accordingly examined the physico-chemical properties and gene expressions in the major ampullate (MA) of *B*. *longinqua* from Sydney and Montevideo to establish whether property variability in this silk is a facilitator of the spider’s invasive success.

## Results

**Figure 2:**
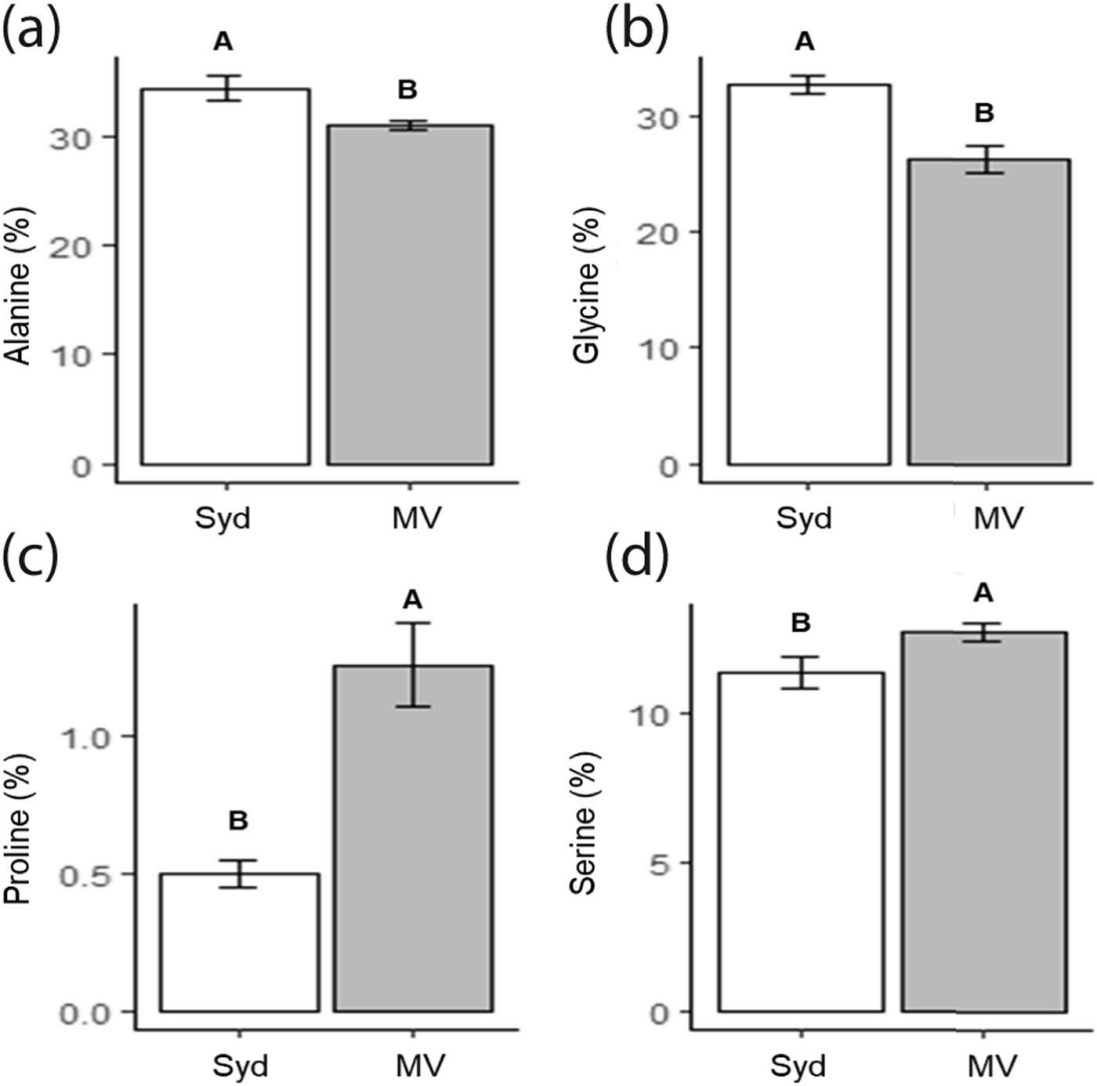
Comparisons of the compositions of (**a**) alanine, (**b**) glycine, (**c**) proline, and (**d**) serine, in the dragline silks of *B*. *longinqua* from Sydney and Montevideo. See Table 1 for statistical comparisons of mean values. Bars represent mean values, whiskers are confidence intervals ±1 s.e.

**Figure 3:**
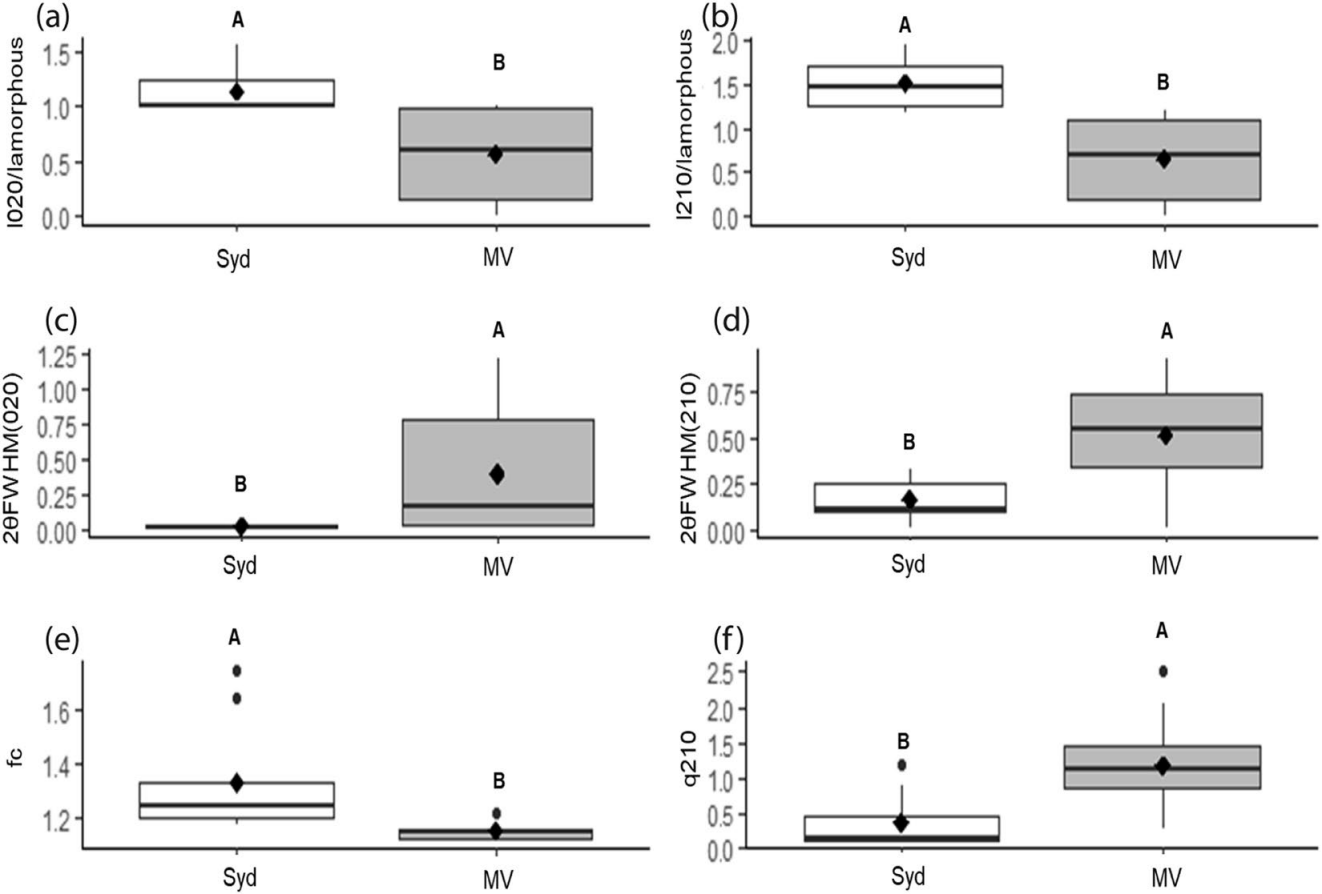
Box-whisker plots comparing the WAXS ascertained nanostructure parameters: (**a**) *I*_020_/*I*_amorphous_, (**b**) *I*_210_/*I*_amorphous_, (**c**) 2*θ* FWHM (0 2 0), (**d**) 2*θ* FWHM (2 1 0), (**e**) f_c_, and (**f**) *q* (2 1 0), derived from WAXS experiments of the dragline silks of *B*. *longinqua* from Sydney and Montevideo. Plots display the median (line), mean (black diamonds), 25% quartiles (boxes), extremities (whiskers), and outliers (peripheral dots). See Table 2 for statistical comparisons of mean values.

**Figure 4:**
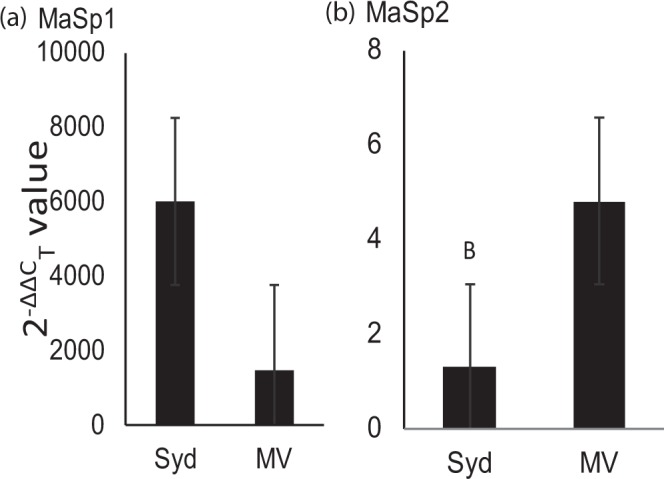
Comparisons of the relative expressions (2^−ΔΔCT^) of (**a**) *MaSp1* and (**b**) *MaSp2* genes between the *B*. *longinqua* Sydney (Syd) and Montevideo (MV) populations. *Indicates that a significant difference was determined by univariate ANOVA. Error bars show ± 1 s.e.

We performed High Performance Liquid Chromatography, wide angle X-ray scattering, and gene expression analyses on the dragline silk of *B*. *longinqua* from Sydney and Montevideo and found locational differences in amino acid compositions (Wilk’s λ = 0.128, df = 4,15, *P* < 0.0001), nanostructures (Wilk’s = 0.021, df = 4,15, *P* = 0.006), and silk protein (spidroin) expressions (Wilk’s = 0.060, df = 4,15, *P* = 0.012). These results suggest there were differential expressions of the proteins major ampullate spidroin 1 (MaSp1) and major ampullate spidroin 2 (MaSp2). Subsequent univariate ANOVAs found that silk glutamine, serine, proline, glycine, and alanine compositions all significantly differed between the Sydney and Montevideo *B*. *longinqua* populations (Table 1; Fig. 2). Likewise, the WAXS ascertained that the parameters signifying silk nanostructure crystallinity, intensity and orientation (*I*_020_*/I*_amorphous_, *I*_210_/*I*_amorphous_, 2*θ* FWHM (0 2 0), 2*θ* FWHM (2 1 0), f_c_, and *q* (2 1 0)) significantly differed between the populations (Table 2; Fig. 3). Our genetic expression analysis found expression of the major ampullate spidroin 2 (*MaSp2*) gene (we use italicisation herein to distinguish the genes from the protein) to differ across populations (F = 3.453, df = 1,18, *P* = 0.043; Fig. 4a), while the expression of major ampullate spidroin 1 (*MaSp1*) gene did not (F_1,18_ = 2.431, df = 1,18, *P* = 0.123; Fig. 4b).

**Table 1:** Results of balanced sequential univariate (one-way) ANOVAs to individually compare the compositions of glutamine, serine, proline, glycine, and alanine in the dragline silks of Australian and Uruguayan *B*. *longinqua*.

		SS	df	MS	F	P
%Alanine	Intercept	21296.83	1	21296.83	29175.00	<0.0001
	Site	57.38	1	57.38	78.61	<0.0001*
	Error	13.14	18	0.73		
%Glycine	Intercept	17357.79	1	17357.79	18308.76	<0.0001
	Site	209.24	1	209.24	220.70	<0.0001*
	Error	17.07	18	0.95		
%Proline	Intercept	15.155	1	15.15	1186.90	<0.0001
	Site	3.057	1	3.057	239.47	<0.0001*
	Error	0.229	18	0.012		
%Serine	Intercept	2913.84	1	2913.84	15756.63	<0.0001
	Site	9.06	1	9.06	49.01	<0.0001*
	Error	3.32	18	0.18		

* indicates significant a difference was found (at P < 0.05).

**Table 2:** Results of balanced sequential univariate (one-way) ANOVAs to individually compare the WAXS determined nanostructure parameters: *q* (2 1 0), 2*θ* FWHM (0 2 0), 2*θ* FWHM (2 1 0), 2*θ* FWHM (amorphous halo), *I*_210_/*I*_amorphous_, *I*_020_/*I*_amorphous,_ and f_c_ in the dragline silks of Australian and Uruguayan *B*. *longinqua*.

		SS	df	MS	F	P
*q*(210)	Intercept	31.04	1	31.04	1502.77	<0.0001
	Site	0.16	1	0.16	7.77	0.012*
	Error	0.37	18	0.02		
*q*(020)	Intercept	42.20	1	42.20	90260.26	<0.0001
	Site	0.01	1	0.01	0.32	0.58
	Error	0.01	18	0.004		
2*θ* FWHM(210)	Intercept	2.33	1	2.33	49.99	<0.0001
	Site	0.59	1	0.59	11.89	0.002*
	Error	0.89	18	0.04		
2*θ* FWHM(020)	Intercept	0.87	1	0.87	8.71	0.008
	Site	0.71	1	0.71	7.11	0.015*
	Error	1.80	18	0.10		
2*θ* FWHM (amorphous)	Intercept	6.88	1	6.88	15.25	0.001
	Site	2.73	1	2.73	6.06	0.02*
	Error	8.11	18	0.45		
*I*_210_/*I*_amorphous_	Intercept	23.51	1	23.51	141.99	<0.0001
	Site	3.71	1	3.71	22.45	<0.0001*
	Error	2.97	18	0.16		
*I*_020_/*I*_amorphous_	Intercept	14.61	1	14.61	126.52	<0.0001
	Site	1.66	1	1.66	14.38	0.001*
	Error	2.07	18	0.11		
X_c_	Intercept	0.09	1	0.09	0.02	0.88
	Site	10.21	1	10.21	2.11	0.16
	Error	87.15	18	4.84		
f_c_	Intercept	6.89	1	6.89	15.91	<0.0001
	Site	3.06	1	3.06	7.08	0.01*
	Error	7.80	18	0.43		

*indicates significant a difference was found (at P < 0.05).

**Figure 5:**
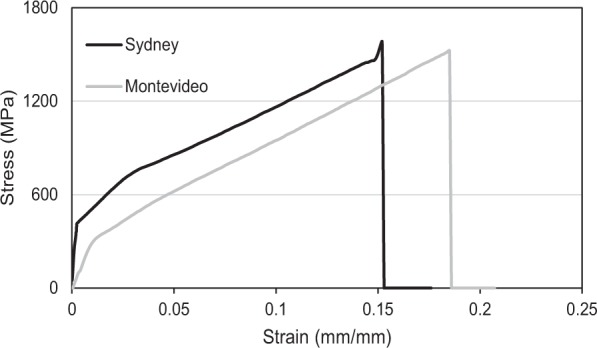
Stress versus strain curves averaged across individual spider and individual thread replicates for the Sydney and Montevideo *B*. *longinqua* populations.

Despite finding significant differences in silk amino acid compositions and nanostructures between populations, we did not find any corresponding differences in the mechanical properties (Wilk’s λ = 0.6277; df = 4,15, *P* = 0.1153, see Fig. 5 for averaged stress versus strain curves for silks from the spiders of each population, and Supplementary Fig. S3 for the comparisons of each of the properties).

## Discussion

### Silk amino acid compositions, protein nanostructures, and gene expression differed between populations

Our finding that silk amino acid compositions, protein nanostructures and gene expressions differed between populations of *B*. *longinqua* from Sydney and Montevideo indicate that the silks produced by spiders at the two sites likely differed in their relative compositions of MaSp1 and MaSp2 proteins with a greater relative expression of MaSp2 among spiders from the Montevideo population. The MaSp2 protein has residues containing proline and serine whereas the MaSp1 protein lacks these residues^22^. The difference in *MaSp2* expression thus explains the greater proline and serine compositions in the silks of the spiders from Montevideo. The presence of the MaSp1 protein likely promotes β–sheet formations in dragline silk fibers while the proline and serine in MaSp2 likely induces the formation of β-turns, 3_10_-helices and other amorphous nanostructures^23,29^. We therefore expect that the greater MaSp1 composition in the silks from the spiders from Sydney, compared those from Montevideo, explains why we found greater X-ray scattering from the crystalline nanostructures of these silks but weaker scattering from their amorphous nanostructures (as exemplified by the *I*_020_/*I*_amorphous_ and *I*_210_/*I*_amorphous_ values shown in Fig. 3). An alternative explanation is that sequence variations, for instance deletions or insertions of tri-nucleotides, might have led to different sized and structured repetitive units and, accordingly, different structures within specific silk proteins^34^.

While our amino acid composition and gene expression analyses showed that there were differences in *MaSp2* expression across the two populations, the *MaSp2* expressions found for spiders from both populations were considerably low compared with *MaSp1* expressions in both populations (see Fig. 4). The MaSp2 protein is thought to be predominant in the silks of many orb web building spiders^35^ (but see Bittencourt *et al*.^36^). However, *B*. *longinqua* belongs to the distantly related family Desidae^17^. While the low and variable *MaSp2* expressions that we found could be attributed to our RT-PCR procedures amplifying the *MaSp* paralogs differently and subsequently reducing the apparent expression of *MaSp2* in some samples, our finding of around 0.5% proline in the silks of spiders from Sydney and around 1.3% proline in the silks of spiders from Montevideo complement our finding that the spiders from both populations consistently expressed relatively low quantities of *MaSp2* compared to *MaSp1*^29,37^.

### Silk mechanical properties were robust between populations despite physico-chemical variation

It is conventionally thought that MaSp1 primarily forms crystalline β–sheet nanostructures, which are responsible for inducing high strength in spider dragline silk, while MaSp2 primarily forms into β-turns and other amorphous nanostructures and induces high silk elasticity^22,38,39^. Our finding that the supposed genetically determined silk amino acid compositional and nanostructural differences between the Sydney and Montevideo *B*. *longinqua* populations did not correspond with any differences in mechanical properties thus seems counterintuitive according to conventional reasoning.

Spider dragline silk mechanical properties have been shown to, on occasions, remain robust despite significant physico-chemical variations in their constituent proteins. For instance, diets that vary in protein content induce nanostructural changes in the dragline silks of the spider *Nephila pilipes* without significant direct effect on the silk’s mechanical properties^30^. We accordingly concluded herein that the mechanical properties of *B*. *longinqua*’s silks seem to be robust despite differences in protein compositions and nanostructures between the silks of spiders from the two populations. We do not know, offhand, the physical means by which the silks retain their mechanical robustness despite significant variability in the protein composition and structure. We, however, predict, based on various lines of evidence in the literature^40–42^, that the mechanical robustness of spider silk can be retained across environments provided any changes to the crystalline and amorphous region proteins do not affect fracture propagations across the skin and/or core domains, or induce any other surface or internal flaws.

Closer examinations of the variability in the amino acid (Fig. 2), nanostructure (Fig. 3), gene expression (Fig. 4), and mechanical property (Supplementary Fig. S3) data reveal some interesting trends. For instance, higher variability in the silk amino acid composition and nanostructure parameters coincided with higher variability in ultimate strength, Young’s modulus, and toughness in the silks from spiders from Montevideo. The Sydney population on the other hand seems to have a consistently low variability in both its physico-chemical and mechanical properties. These across population trends suggest that silk physico-chemical properties are more variable for spiders from the Montevideo population than the Sydney population.

### Can variability in silk properties contribute to *B*. *longinqua*’s invasive success?

Given the abovementioned results, we concluded that the silks of the spiders from the Sydney population were not as variable across parameters as those from spiders from the Montevideo population, at least among the spiders we sampled. This might be because the diet and habitats utilized by the spiders in urban Sydney were particularly homogeneous compared to those at Montevideo. This supposition nevertheless warrants further investigation for a larger number of spiders. If it can be substantiated it might have profound implications for understanding and estimating the global invasibilities of many spiders.

Since we collected all of our spiders from urban trees, walls, and windows, in spring, the microhabitats that we sampled from were considered identical across our Montevideo and Sydney sites. We nonetheless realistically expect there to have been broader scale differences between the sites that might contribute towards differences in the silks from the two populations. Relative humidity is markedly higher in Montevideo compared to Sydney all year round (see Supplementary Fig. S2b), and this likely induces significant differences in the type and abundance of insect assemblages at the two locations. We have seen *B*. *longinqua* frequently capture and consume ants, small flies, and beetles, at both locations during our sampling. However, it is certainly possible that different species of insects are encountered throughout the year at each site. The most significant behavioural and dietary difference that we noticed between sites, nonetheless, was spiders within trees in Montevideo invading and consuming colonies of the subsocial spider *Anelosimus vierae* (see Supplementary Fig. S1), whereas the spiders in trees in Sydney consumed just ants, flies and beetles. Given diet is a strong mediator of differential silk gene expression and can induce significant physico-chemical and nanostructural property variations in spider silk^29,30,43^, we expect that dietary differences between the populations is a likely mediator of the differences in the physico-chemical properties of *B*. *longinqua* dragline silk. We, nevertheless, need direct experimental verification of this expectation.

We expect that a lack of aggression and year-round activities render *A*. *vierae* vulnerable to attacks by *B*. *longinqua*. Regardless of the reason, the invasion of *A*. *vierae* colonies by *B*. *longinqua* in Montevideo represents a significant shift in foraging behaviour in *B*. *longinqua* compared to the native population from Sydney. Moreover, feeding on spiders induces a significant shift in the trophic niche^44^ occupied by *B*. *longliqua* in Montevideo compared to Sydney. Such changes may further facilitate or induce dragline silk physico-chemical plasticity. Experiments are needed to reveal the proximal mechanisms, but we expect that shifts in the behavioural and/or trophic niche are likely responsible for the greater physico-chemical property variation we found in the *B*. *longinqua* silk from Montevideo.

Spiders extract different quantities of nutrients when they consume invertebrate predators, especially when those predators are other spiders, than when they consume invertebrate herbivores^45,46^. Accordingly, if *B*. *longinqua* are feeding more on other spiders than insects in Montevideo the nutrients available to them for investment in the different silk proteins would be affected^46^. We thus think it is reasonable to predict that diet could be the factor most responsible for the differences in spidroin expression between populations. Furthermore, the building of webs on or near *A*. *vierae* colonies, and the invasion of the colonies to exploit the spiders within, represents a significant shift in the use and application of webs and silks by *B*. *longinqua* in Montevideo. Elsewhere *B*. *longinqua* builds webs to passively ensnare prey that fly or crawl nearby^14^. We might accordingly expect that such a shift in the use of webs and silks by the spiders in Montevideo requires the production of silks with different functional properties compared to those used elsewhere^47^. Nevertheless, the mechanical properties of the silks of spiders from Montevideo were comparable to those of spiders from Sydney so it seemed that, in contrast to our expectations, the different webs and silks used across the populations did not necessitate the production of silks with different mechanical properties.

There is good evidence to suggest that variability in plant or animal phenotypes influence their invasibilities by expediting rapid physiological adaptations across new environments^6,8,10^. We herein found evidence that variability in an extended phenotype, i.e. spider silks and webs, might facilitate the successful intercontinental invasibility of the spider *Badumna longinqua* and, quite expectantly, other spiders. It is certainly feasible that variability in certain extended phenotypes and structures could facilitate niche construction and ecosystem engineering effects that promote novel adaptations^20,48,49^, which would further extenuate the invasibilities of some animals. It is of high conservation importance that we continue to analyse the potential factors promoting the establishment and growth of invasive species as they can negatively affect endemic populations. This appears to have occurred in Uruguay where we have seen the size and abundance of *A*. *vierae* colonies to become significantly diminished over the past 10 years or so, all the while *B*. *longinqua* has become more prevalent.

## Conclusions

Here we examined the physico-chemical properties and genetic expression of dragline silk from Australian (i.e. the native population) and Uruguayan (i.e. the invasive population) populations of *Badumna longinqua* to assess whether MA silk property variability is a facilitator of its invasive success. Our subsequent analyses found variations in *B*. *longinqua*’s silk amino acid compositions, protein nanostructures, and silk gene (spidroin) expressions. Nevertheless, we did not find any differences in the mechanical properties of its dragline silks between populations. These results indicate that differences in the silk genes expressed by spiders in the two populations manifests as changes in amino acid composition and protein nanostructures in their dragline silks. Nevertheless, *B*. *longinqua*’s silk retained its functional integrity despite significant variability in its physico-chemical properties. We subsequently predict that retaining the functional integrity of its MA silk as the physico-chemical properties vary plastically enables *B*. *longinqua* to become an exceptionally successful invader. Nevertheless, confirmatory tests of this prediction are required. We are continuing our monitoring program of the spider in both Montevideo and Sydney to assess if the two *B*. *longinqua* populations truly differ in their feeding behaviours, thus diets, and to monitor whether its presence has detrimental impacts on *A*. *vierae* populations in Montevideo.

Broadly, our findings indicate that spider silk genetic and subsequent protein expression is not necessarily an indicator of its mechanical properties and, conversely, that the stress versus strain properties alone cannot reveal the underlying genetic and protein compositions in a particular silk.

## Methods

### Spiders and locations

Individual adult female *B*. *longinqua* were collected from randomly selected locations around urban Sydney, Australia, and Montevideo, Ururguay, during mid-spring (i.e. October-November) 2015. We collected 10 spiders of similar length and mass from both sites (Uruguay: body length = 13.814 ± 0.509 mm, mass = 0.316 ± 0.075 g; Australia: body length = 13.654 ± 0.468 mm, mass = 0.324 ± 0.107 g, as determined from referenced digitized photographs and electronic balance, respectively). Spiders were collected predominantly from trees, fences, walls, and windows, on the outside of buildings and other urban structures at both sites. We accordingly considered the microhabitats occupied by the spiders sampled at each site to be structurally and thermally identical.

### Collection of silk

Upon collection we immediately brought the spiders to the laboratory at either the University of New South Wales (Sydney) or Ecología del Comportamiento (Montevideo). We then anaesthetized the spiders using carbon dioxide and placed each of them ventral side up on a 150 mm × 100 mm foam platform and immobilized their legs using non-adhesive tape and pins before carefully extruding a single dragline silk fiber from the major ampullate spigot (identified under a dissecting light microscope at x10 magnification) using tweezers. Since the anaesthetic procedures were tightly controlled we were confident that anaesthesia alone did not affect any subsequently measured silk properties. The silk fibers were collected for the following amino acid composition, tensile testing, and wide-angle X-ray scattering (WAXS) analyses by a mechanical spool spun at a constant speed (1 m min^−1^) under controlled temperature (25 ± 1 °C) and humidity (50 ± 5% R.H.) in still air (see Blamires *et al*.^29,50^; Lacava *et al*.^47^; Benamú *et al*.^51^, and below, for additional details).

### Amino acid composition analysis

To collect silk for amino acid composition analyses we spooled from each spider a single MA silk fiber around a glass cylindrical tube (diameter = 1.5 mm). The silks were scraped off the tube using a scalpel blade and weighed to the nearest 0.001 mg on an electronic balance (Pioneer PA214C, Ohaus, Pine Brook NJ, USA) before being placed into 0.5 ml Eppendorf tubes and sent to the Australian Proteomic Analysis Facility, Sydney, Australia.

Spider dragline silk is thought to be composed of two proteins, called MaSp1 (major ampullate spidroin 1) and MaSp2 (major ampullate spidroin 2). Since MaSp1 consists of repeated residues of alanine and glycine, while MaSp2 consists of residues containing proline and serine in addition to alanine and glycine^22^, we measured the compositions of the amino acids alanine, glycine, proline and serine in the dragline silks from each population using High Performance Liquid Chromatography, as described in detail elsewhere^29^.

### Tensile testing

We spooled dragline silk from five individuals by attaching a headframe to the spool and wrapped a 240 mm long cardboard strip with six 10 mm × 10 mm square holes punched at 10 mm intervals around the headframe. Double sided adhesive tape was stuck onto the cardboard bordering the holes. The headframe was rotated once ensuring the silk traversed all of the holes and adhered to the tape. The strip was then removed from the headframe and a drop of Elmer’s glue applied to the position where the silk was fastened to the cardboard. Another frame of equal size with identically positioned holes punched into it was placed on top. The two strips were squeezed together with forceps ensuring that they stuck together. We then cut the strip at the regions between the holes perpendicular to the silk thread, leaving six 10 mm × 10 mm frames holding a single 10 mm length of silk thread for each spider (see Blamires *et al*.^29,50^; Benamú *et al*.^51^ for details).

One frame of silk collected from each spider was used to ascertain the width of the thread to account for the cross-sectional area in the ensuing tensile tests by taping the frame to a microscope slide and examining and photographing it under 400x magnification using a polarized light microscope (CKX41, Olympus, Tokyo, Japan) connected to a SPOT Idea 5 Mp digital camera (Spot Imaging Solutions, Sterling Heights, MI, USA). The images were digitized using the program Spot Basic 4.7 (Spot Imaging Solutions) and the width of each thread determined as a mean of 12 measurements using Image J (NIH, Bethesda, MD, USA).

For the remaining 50 frame-mounted silk samples (5 each from 5 individuals across the two locations) we performed the following tensile tests. We first placed the 10 mm × 10 mm frames containing a single fiber within the grips of a T150 (Agilent Technologies, Santa Clara, CA, USA) nano-tensile testing machine. We ensured that the grips held the silks firmly at the upper and lower frame edges. The left and right sides of the frames were then cut away and the silks were stretched at a rate of 0.1 mms^−1^ until the fiber ruptured.

Stress (σ) and strain (ε) were calculated using equations:

1 $${\rm{\sigma }}=\frac{{\rm{F}}}{{\rm{A}}}$$

and

2 $${\rm{\varepsilon }}={\log }_{{\rm{e}}}\frac{{\rm{L}}}{{{\rm{L}}}_{0}}$$

where F is the force applied to the specimen measured using the program Nano Suite 1.0 (Agilent Technologies, Santa Clara CA, USA), and A is the cross-sectional area of the thread calculated from the thread diameter assuming a constant thread volume^40,52^. L is the instantaneous length of the fiber at a given extension value, measured using Nano Suite, and L_0_ is the original gage length of the fiber (i.e. 10 mm).

Stress versus strain curves were plotted for each silk tested from which we calculated the following mechanical properties for comparisons between the Sydney and Montevideo populations: (i) ultimate strength; or the stress at rupture, (ii) extensibility; or the strain at rupture, (iii) toughness; the area under the stress strain curve, and (iv) Young’s modulus (an estimate of stiffness); the slope of the stress-strain curve during its initial elastic phase.

### Wide-angle X-ray scattering analyses

The alanine/glycine residues within MaSp1 are expected to promote crystalline β–sheet formations in dragline silk fibers, while the addition of proline and serine in MaSp2 are expected to induce the formation of additional β-turns and amorphous nanostructures^22^. To examine and compare these nanostructures in the dragline silks of Australian and Uruguayan *B*. *longinqua* populations, we performed wide-angle X-ray scattering analyses at the SAXS/WAXS beamline at Australian Synchrotron, Melbourne, Australia.

In our Sydney and Montevideo laboratories we collected silk from each spider on individually constructed 3 mm × 1 mm steel frames with 0.5 mm × 0.5 mm windows^35^. We pulled the silk threads perpendicularly across the frame window and ran the spool for ~2 h at a constant speed, thus ensuring approximately 2000 rounds of silk were wrapped around the frame windows. At Australian Synchrotron we taped each frame to a sample plate mounted onto a plate holder at a distance of 330 mm from the incident X-ray beam. We exposed each sample to the beam for 10–60 s depending on its density. The photon scattering from each silk sample were detected by a Mar 165 imaging plate (Q-value = 1.45 Å). Two-dimensional WAXS images were subsequently developed using the program Scatterbrain (Version 2.82, Australian Synchrotron, Melbourne, Australia) and examined as follows.

From the images we calculated the: (i) scattering parameter (*q*), (ii) diffraction angles (2*θ*), (iii) diffraction intensity (*I*_x_), and (iv) full width and half width maximum intensities (FWHM) of the 2*θ* angles at the (0 2 0) and (2 1 0) diffraction peaks (the peaks associated with scattering from crystalline β–sheets in silks, see Blamires *et al*.^29,30^) and the amorphous halo, using Scatterbrain. These parameters provided us information about crystal sizes and alignments. We then calculated, for comparisons between treatments:

(i) The relative crystalline to amorphous intensity ratios *I*_020_/*I*_amorphous_ and *I*_210_/*I*_amorphous_ with *I*_020_, *I*_210_ and *I*_amorphous_ representing the sum of the intensity peaks measured for the (0 2 0) and (2 1 0) reflection vectors, and the amorphous region, respectively.

(ii) The crystallinity index, *X*_c_, as^53^:

3 $${X}_{c}=(({I}_{020}-{I}_{210})/({I}_{020}))\times 100$$

(iii) Herman’s orientation function, f_c_, using the equation^53^:

4 $${{\rm{f}}}_{{\rm{c}}}=(3\{{\cos }^{2}{\rm{\phi }}\rangle \}-1)/2$$

where φ is the angle between the c axis and the fiber axis, {cos^2^φ} is the azimuthal width of the two strongest equatorial reflections (020) and (210), determined using the equation:

5 $$\{\cos \,2{\rm{\phi }}\}={\rm{1}}-{\rm{A}}\{\cos \,2{\rm{\phi }}1\}-{\rm{B}}\{\cos \,2{\rm{\phi }}2\}$$

where A = 0.8 and B = 1.2.

### RT-qPCR analyses of spidroin expressions

Upon silk collection, all of the spiders were sacrificed by lethal exposure to CO_2_ and their major ampullate glands identified and dissected as described by Jeffrey *et al*.^54^. The glands and a small sample of remaining abdomen were immediately flash frozen at −80 °C. The samples taken in Uruguay were transported on liquid nitrogen to Sydney while the samples taken in Sydney were stored flash frozen, whereupon the following mRNA extractions, reverse transcriptions and RT-qPCR analyses were performed to compare the expressions of *MaSp*1 and *MaSp*2 genes between the Australian and Uruguayan populations.

The silk gland and abdomen samples were lysed with RNase free mini pestles in QIAzol Lysis Reagent and mRNA extracted using an RNeasy Plus Universal RNA extraction kit (Qiagen, Düsseldorf, Germany). To prevent any genomic DNA contamination we used a gDNA Eliminator Solution provided with the extraction kit. The extracted mRNA was eluted to 30–35 μl and we measured the concentration extracted using a NanoDrop 1000 Spectrophotometer (Thermo Fisher Scientific, Wilmington, DE, USA). A mean concentration of 634.24 ± 122.24 ng/μl RNA was extracted from all glands and a mean concentration of 698.94 ± 90.44 ng/μl RNA was extracted from all abdomens, with no differences between the Sydney and Montevideo populations (glands: *t* = 0.542, df = 8, *P* = 0.616, abdomens: *t* = 2.412, df = 8, *P* = 0.086). The mean absorbance ratios of samples from silk glands of the Sydney spiders were 1.68 at 260/280 nm, and ranged between 1.24 and 2.20, and 2.10 at 260/230 nm (ranging between 1.91 and 2.19). The mean absorbance ratios of the samples from the silk glands of the Montevideo spiders were 2.10 at 260/280 nm, and ranged between 2.00 and 2.13, and 1.83 at 260/230 nm (ranging between 1.74 and 2.04). We considered these absorbance ratios as acceptably pure samples of single stranded RNA for our subsequent analyses.

All extracted mRNA was diluted to 100 ng/μl, from which we took 12.5 μl aliquots for reverse transcription to cDNA using an Advantage reverse transcription kit for PCR (Clontech, Clayton Vic, Australia). The reverse transcription and PCR activation procedures were carried out using Eppindorf Mastercycler (Eppindorf, Mamburg, Germany) qPCR machines, following the recipe outlined within the reverse transcription kit handbook. A “buffers only” (i.e. no RNA/no Reverse Transcriptase) solution was included in the analyses as a negative control. We included the spider’s abdomens in the gene expression analyses for normalization against background *MaSp1*/*MaSp2* transcripts from other silk glands and/or abdominal tissue. All procedures were replicated three times for each individual spider.

We used the *Drosophila rufa* glycerol-3-phosphate dehydrogenase (g3pdh) gene as a “housekeeping” reference gene for our RT-PCR analyses, as is common practice in gene expression analyses to standardize the samples and remove any influence of the experimental protocols from the outputs^55,56^. Since the g3pdh gene primers were based on a transcript from a distantly related insect there were some inevitable, albeit minor, amplification biases across samples^55,57^.

We diluted all cDNA eluents to 200 ng/μl, (using a NanoDrop 1000 Spectrophotometer), checking that the 260:280 nm absorbance ratio were all between 1.6 and 1.8 before sending 10 μl samples to the Ramaciotti Centre for Genomics, University of New South Wales, for Fluidigm quantitative RT-PCR gene expression analyses using a Taqman preamp master mix for STA (14 cycles) and SSOFast for the qPCR runs^29,58^.

We used published C-terminal domain sequences for *MaSp*1 and *MaSp*2 derived for *Nephila clavipes*^59,60^ to order primers for the RT-PCR analyses using the Fluidigm online assay designer^57^. We converted the threshold cycle (C_T_) values derived by the RT-PCR analyses to 2^−ΔΔCT^ values, which were averaged for each individual spider across the three technical replicates following Schmittgen & Livak^61^.

### Statistical analyses

We used separate one-way (site: Sydney or Montevideo) multivariate analyses of variance (MANOVAs) to determine whether the: (1) compositions of glutamine, serine, proline, glycine, and alanine, (2) mechanical properties (ultimate strength, extensibility, toughness, and Young’s modulus), (3) nanostructures (*q* and 2*θ* FWHM of the (0 2 0) and (2 1 0) diffraction peaks and amorphous halo, *I*_020_/*I*_amorphous_, *I*_210_/*I*_amorphous,_ X_c_, and f_c_), and (4) *MaSp1* and/or *MaSp2* expressions (2^−ΔΔCT^ values), differed between silks from the Sydney and Montevideo populations. Where MANOVA indicated a significant difference between locations we followed it up with balanced sequential univariate (one-way) ANOVAs to individually compare the mean values for each parameter between sites. Prior to performing analyses we used a combination of Bartlett’s heterogeneity of variances tests, Box’s M tests, cross-correlations, and Q-Q scatterplots to test the ANOVA/MANOVA assumptions, including the homogeneity of variances, normality of error distributions, independence of observations, linearity, absence of co-linearity, and equality of covariance matrices. We log_10_ or arcsine (e.g. amino acid composition data) transformed any data that failed any of these tests.

## Supplementary information

Supplementary Figure S1–S3

## Data Availability

Data supporting the results will be archived in Dryad .
