# Author Correction: Silk physico-chemical variability and mechanical robustness facilitates intercontinental invasibility of a spider

**DOI:** 10.1038/s41598-019-54237-4

**Published:** 2019-11-27

**Authors:** Carmen Viera, Luis F. Garcia, Mariángeles Lacava, Jian Fang, Xungai Wang, Michael M. Kasumovic, Sean J. Blamires

**Affiliations:** 1 Entomología, Universidad de la República de Uruguay, Montevideo, Uruguay; 2 Laboratorio Ecología del Comportamiento (IIBCE), Montevideo, Uruguay; 3 Centro Universitario Regional del Este, Sede Treinta y Tres. Universidad de la República, Treinta y Tres, Uruguay; 4 Centro Universitario de Rivera, Universidad de la República, Rivera, Uruguay; 5 Deakin University, Institute for Frontier Materials (IFM), Waurn Ponds Campus, Geelong, 3220 Australia; 6 Evolution & Ecology Research Centre, School of Biological, Earth & Environmental Sciences, The University of New South Wales, Sydney, NSW 2052 Australia

Correction to: *Scientific Reports* 10.1038/s41598-019-49463-9, published online 13 September 2019

In the original version of this Article, Luis F. Garcia was incorrectly affiliated with ‘Laboratorio Ecología del Comportamiento (IIBCE), Montevideo, Uruguay’, ‘Ciencias Biológicas PEDECIBA, Docente Grado 2 Entomología, CURE, Treinta y Tres, Uruguay’ and ‘Centro Universitario de Rivera, Universidad de la República, Rivera, Uruguay’.

The correct affiliation is listed below.

Centro Universitario Regional del Este, Sede Treinta y Tres. Universidad de la República. Treinta y Tres, Uruguay.

This error has now been corrected in the PDF and HTML versions of the Article and in the accompanying Supplementary Figure S1–S3 file.

