# Supplementary material for: Silk physico-chemical variability and mechanical robustness facilitates intercontinental invasibility of a spider
Source: Sci Rep. 2019 Sep 13;9:13273. doi: 10.1038/s41598-019-49463-9 (PMC6744404; doi:10.1038/s41598-019-49463-9)
Supplement: Supplementary file 1 — Supplementary Figure S1–S3 [file 41598_2019_49463_MOESM1_ESM.docx]

**Silk physico-chemical variability and mechanical robustness facilitates intercontinental invasibility of a spider**

Carmen Viera^1,2^, Luis F. Garcia^3^, Mariángeles Lacava^2,4^, Jian Fang^5^, Xungai Wang^5^, Michael M. Kasumovic^6^, Sean J. Blamires^6^

# ^1^Entomología,[Universidad de la República de Uruguay](https://www.researchgate.net/institution/Universidad_de_la_Republica_de_Uruguay), Montevideo, Uruguay

^2^Laboratorio Ecología del Comportamiento (IIBCE), Montevideo, Uruguay

^3^Centro Universitario Regional del Este, Sede Treinta y Tres. Universidad de la República. Treinta y Tres, Uruguay.

^4^Centro Universitario de Rivera, Universidad de la República, Rivera, Uruguay

^5^Deakin University, Institute for Frontier Materials (IFM), Waurn Ponds Campus, Geelong 3220, Australia

^6^Evolution & Ecology Research Centre, School of Biological, Earth & Environmental Sciences, The University of New South Wales, Sydney, NSW 2052, Australia


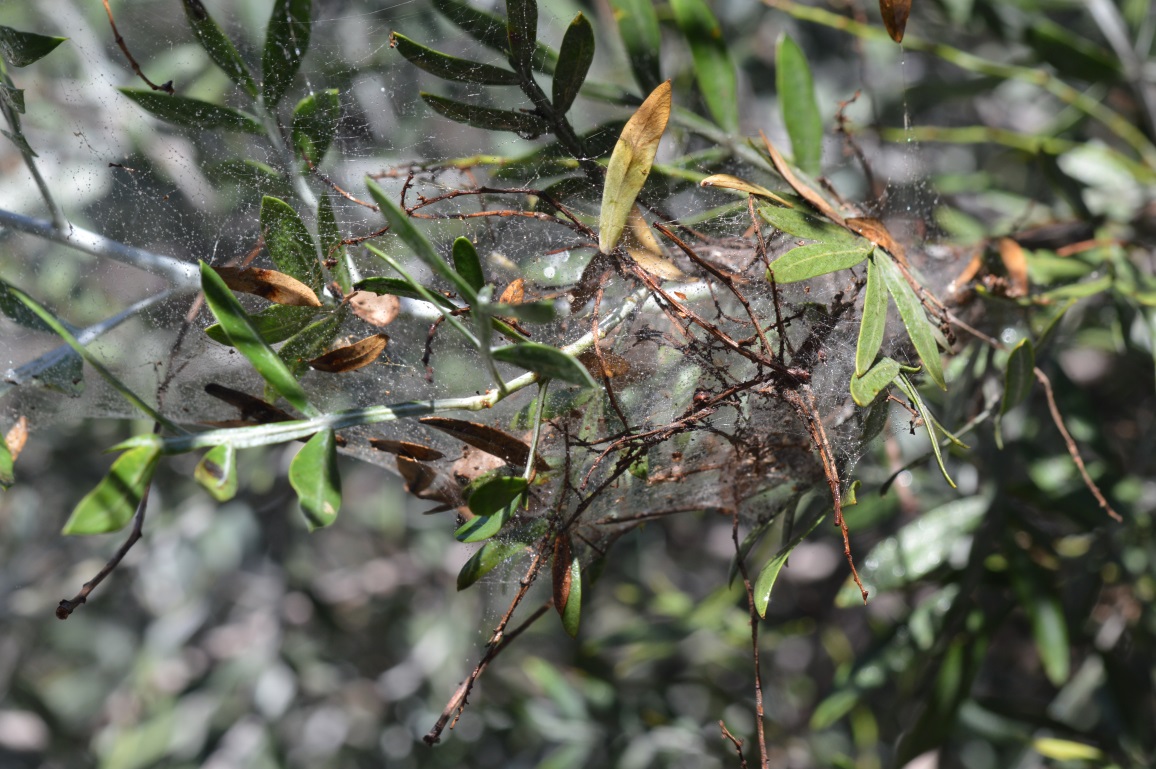


**(a)**


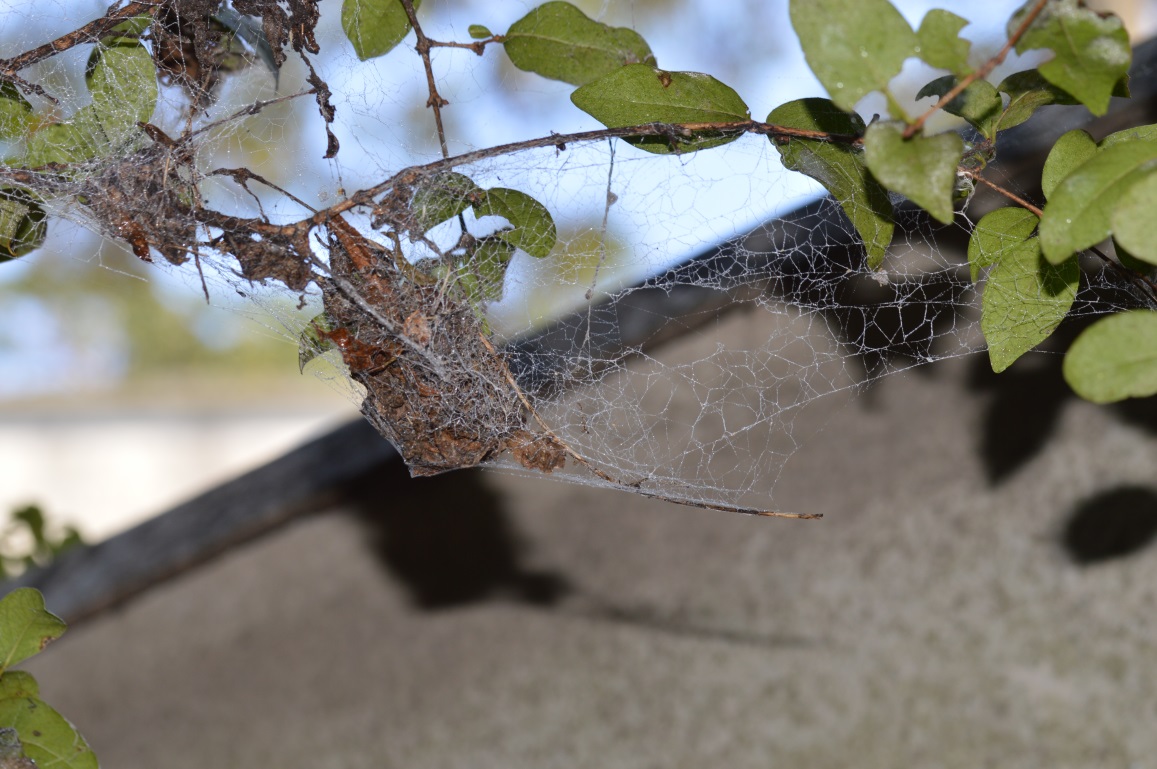


**(b)**

**Figure S1.** Photographs of (A) *Anelosimus vierae* colony from Montevideo without invasion by *Badumna longinqua*, and (B) *Anelosimus vierae* colony from Montevideo after invasion by *Badumna longinqua* (all photographs by S. Blamires).

(a)

(b)

**Figure S2.** Comparisons of average monthly (A) maximum and minimum temperatures and (B) relative humidities between Sydney and Montevideo.


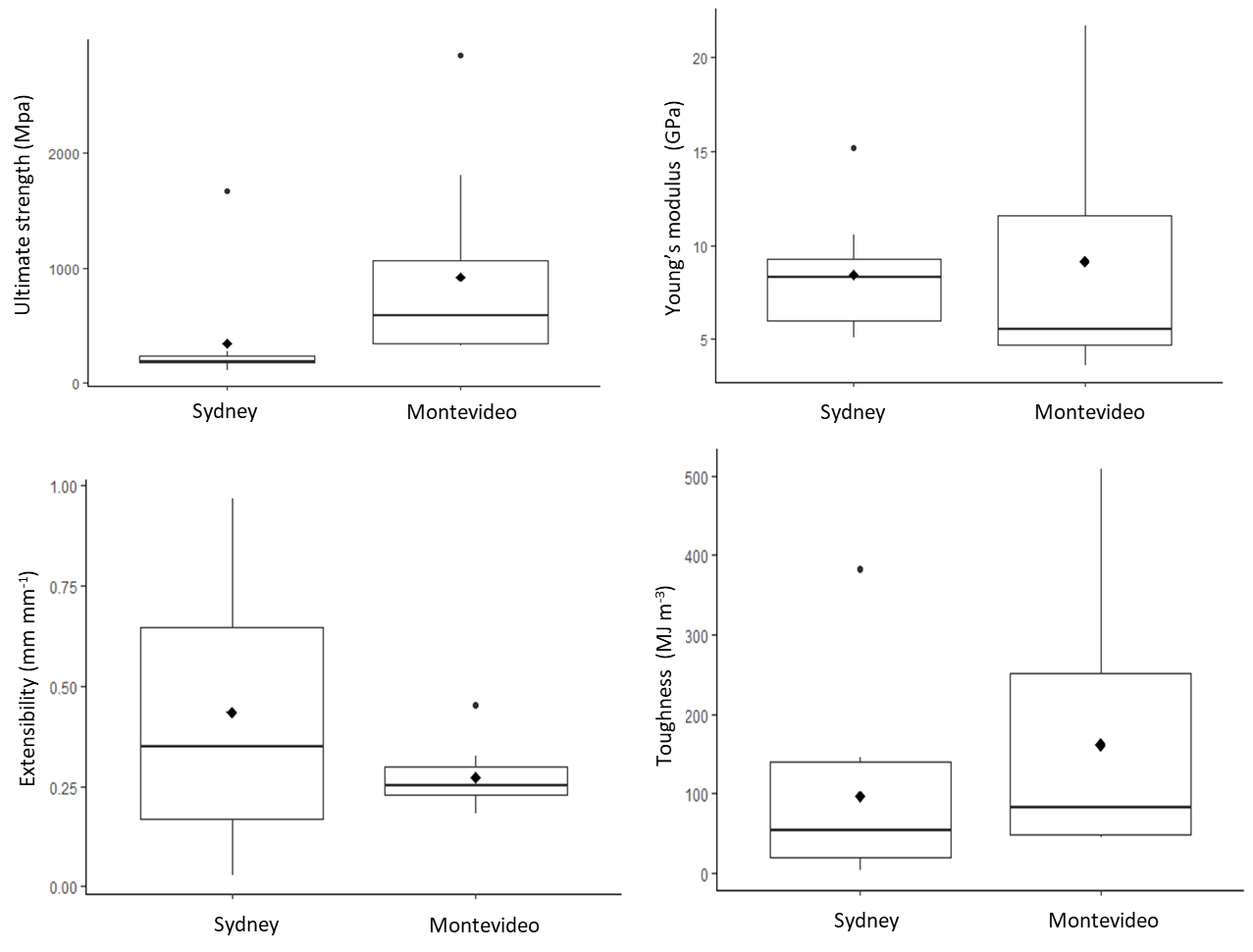


**Figure S3.** Box-whisker plots comparing the mechanical properties Ultimate strength, Extensibility, Young’s modulus, and Toughness, of the dragline silks of *B. longinqua* from Sydney and Montevideo. See Fig. 2 for comparison of the typical silk stress versus strain curves.
